# Modelling the risk of being bitten by malaria vectors in a vector control area in southern Benin, west Africa

**DOI:** 10.1186/1756-3305-6-71

**Published:** 2013-03-15

**Authors:** Nicolas Moiroux, Abdul S Bio-Bangana, Armel Djènontin, Fabrice Chandre, Vincent Corbel, Hélène Guis

**Affiliations:** 1 MIVEGEC (IRD 224-CNRS 5290-UM1-UM2), Institut de Recherche pour le Développement (IRD), BP64501, Montpellier, 34394, France; 2 MIVEGEC (IRD 224-CNRS 5290-UM1-UM2), Institut de Recherche pour le Développement (IRD), Cotonou, 01 BP4414 RP, Bénin; 3 Centre de Recherche en Entomologie de Cotonou (CREC), Ministère de la Santé, Cotonou, Bénin; 4 Department of Entomology, Faculty of Agriculture, Kasetsart University, Bangkok, 10900, Thailand; 5 CIRAD, UMR CMAEE, Montpellier, F-34398, France; 6 INRA, UMR 1309 CMAEE, Montpellier, F-34398, France

**Keywords:** Malaria, Anopheles, Remote sensing, Modelling, Host-vector contact

## Abstract

**Background:**

The diversity of malaria vector populations, expressing various resistance and/or behavioural patterns could explain the reduced effectiveness of vector control interventions reported in some African countries. A better understanding of the ecology and distribution of malaria vectors is essential to design more effective and sustainable strategies for malaria control and elimination. Here, we analyzed the spatio-temporal risk of the contact between humans and the sympatric *An. funestus* and both M and S molecular forms of *An. gambiae s.s.* in an area of Benin with high coverage of vector control measures with an unprecedented level of resolution.

**Methods:**

Presence-absence data for the three vectors from 1-year human-landing collections in 19 villages were assessed using binomial mixed-effects models according to vector control measures and environmental covariates derived from field and remote sensing data. After 8-fold cross-validations of the models, predictive maps of the risk of the contact between humans and the sympatric *An. funestus* and both molecular M and S forms of *An. gambiae s.s.* were computed.

**Results:**

Model validations showed that the *An. funestus*, *An. gambiae* M form, and S form models provided an excellent (Area Under Curve>0.9), a good (AUC>0.8), and an acceptable (AUC>0.7) level of prediction, respectively. The distribution area of the probability of contact between human and *An. funestus* largely overlaps that of *An. gambiae* M form but this latter showed important seasonal variation. *An. gambiae* S form also showed seasonal variation but with different ecological preferences. Landscape data were useful to discriminate between the species’ distributions.

**Conclusions:**

These results showed that available remote sensing data could help in predicting the human-vector contact for several species of malaria vectors at a village level scale. The predictive maps showed seasonal and spatial variations in the risk of human-vector contact for all three vectors. Such maps could help Malaria Control Programmes to implement more effective vector control strategy by taking into account to the dynamics of malaria vector species.

## Background

In response to the worldwide decrease in deaths due to malaria [1,2], malaria elimination is back on the global agenda [3]. However, evidences of reduced effectiveness of vector control interventions have been reported in some African countries [4-6], hence challenging confidence in malaria control efforts in the region. According to the authors of these studies, the diversity of malaria vector populations, expressing various resistance and/or behavioural patterns could explain this trend. A better understanding of the mosquito ecology and distribution is thus essential to take into account this diversity and to design more effective and sustainable strategies for malaria control and elimination [7-9].

The ~60 species of *Anopheles* which are considered as vectors of malaria [10] vary in terms of their capacity to transmit the disease because of variations in their susceptibility to the parasite (vectorial competence), host-seeking preferences (anthropophagy), and/or abundance (this latter being related to the local availability of their specific habitat) [11]. The difference in vectorial capacity can also be observed at the sub-species level. Indeed, M and S molecular forms of *An. gambiae s.s.* (sister taxa that are under speciation process [12]) have different ecological preferences [13-15] and susceptibility to the *Plasmodium* infection [16]. Regarding their ecological preferences, the S form was more frequently observed in the driest areas, in open fields that accommodate for temporary and rainfall-dependent breeding sites with low predation [13,14,17] whereas the M form showed a longer larval development [18,19] and a low sensibility to predation [20,21] highlighting a better adaptation to more permanent breeding sites. Moreover, major malaria vector species facing the wide use of residual insecticides in Africa have adapted by expressing different “strategies” including insecticide resistance [22] or behavioural avoidance [23-25]. These strategies seem to vary according to the vector population and/or the species [22,26-33].

In the Ouidah-Kpomasse-Tori Bossito (OKT) region in southern Benin, a recent cluster randomized controlled trial (RCT) did not report any benefits of using combined long-lasting insecticidal nets (LLIN) and carbamate indoor residual spraying (IRS) or Carbamate Treated Plastic Sheeting (CTPS) compared to selective coverage of LLIN [5]. In this area, the three major African malaria vectors, *An. gambiae* M and S forms and *An. funestus* are sympatric, with spatial and temporal variations [31]. High levels of physiological resistance have been recorded in both M and S forms of *An. gambiae* populations [34-36] whereas *An. funestus* seems to avoid the contact with the insecticide by modulating its biting behaviour [23]. The presence, in sympatry, of three major malaria vectors with different ecologies and resistance patterns gives a particularly interesting opportunity to study the bioecology of these vectors exposed to vector control interventions.

In the present work, we studied the spatio-temporal risk of the contact between humans and the sympatric *An. funestus* and both M and S molecular forms of *An. gambiae s.s.* according to a set of environmental covariates in 19 villages in southern Benin, with an unprecedented level of resolution. Using Binomial mixed-effect models, we assessed the environmental determinants (including vector control measures) of the probability of human-vector contact and we mapped the predicted risk of being bitten by each of these vectors during the dry and the rainy seasons.

## Methods

### Study area and vector control interventions

This study was carried out in the Ouidah-Kpomassè-Tori Bossito (OKT) health administrative region in southern Benin (on the Atlantic coast). Of the 58 villages screened at the baseline, 30 were excluded because they did not fulfil inclusion criteria *i.e.* distance between two villages greater than two kilometres, population size between 250 and 500 inhabitants with non-isolated habitations, and absence of any local health care centre [5]. Twenty-eight villages were randomly assigned to four groups (seven villages by groups) for implementation of four vector control interventions: (1) targeted LLIN coverage (TLLIN) to pregnant women and children younger than six years; (2) universal LLIN coverage of sleeping units (ULLIN), (3) targeted LLIN coverage plus full coverage of carbamate IRS (TLLIN+IRS), and (4) universal LLIN coverage plus full coverage of carbamate CTPS lined up to the walls of the household (ULLIN+CTPS). Of the 28 villages used during the clinical trial, nine (three TLLIN, two ULLIN, two ULLIN+CTPS, and two TLLIN+IRS) were excluded because they were not covered by the satellite image used for the landscape analysis. Details about the allocation of vector control intervention methods have been described elsewhere [5].

### Mosquito collections

Mosquitoes were collected every six weeks during the year 2009 (eight surveys) using the human landing catch (HLC) technique. HLC were carried out from 22:00 to 06:00 both indoors and outdoors of four houses in each village on two successive nights for each survey (i.e., 16 collector-nights per village per survey). Sites were a minimum distance of 50 metres from each other and were homogeneously distributed across the village (sites situated near eucalyptus trees, smoke, etc. were discarded). Collectors were rotated hourly between collection sites and/or position (indoor/outdoor). Independent staff supervised rotations and regularly checked the quality of mosquito collections.

Malaria vectors collected on humans were identified using morphological keys [37,38]. All mosquitoes belonging to the Gambiae complex and the *Funestus* Group were identified to species by PCR [39,40]. Both M and S molecular forms of *An. gambiae s.s.* were identified by the method of Favia *et al*. [41].

### Peri-domestic breeding sites inventory

Anthropogenic larval habitats inside the village borders were surveyed using a standard dipping method [42]. Sampling was repeated during each survey of adult collection and the number of breeding sites positive (or not) for the presence of *Anopheles sp.* larvae was recorded. The Breteau index, representing the number of positive containers for larvae per 100 houses was estimated in each locality [43].

### Size of the buffer zone

To characterize the environment in the neighbourhood of the 19 villages, buffer zones were defined around each village. According to Service [44], two kilometres is the maximum flight range for *Anopheles sp.* and breeding sites located beyond that distance can be considered as insignificant. The distance of two kilometres was therefore selected as the radius of the buffer zones.

### Spatio-temporal data

We used the Land-Surface Temperature (LST) at a spatial resolution of one kilometre measured by the Moderate Resolution Imaging Spectroradiometer (MODIS) sensors on the Terra satellite (https://lpdaac.usgs.gov/). The weekly nocturnal and diurnal temperatures during the week of the surveys and during each of both weeks preceding the surveys were extracted using ArcGis ArcInfo 9.3 software (ESRI, Redlands, CA) in the buffer zone of each village. When the data were not available at a specific date, we used the TiSEG software [45] to estimate missing data: according to the quality statement of the pixels provided as metadata with MODIS data, pixels that do not fit a "good" or an "acceptable" quality (because of clouds for example) were temporally interpolated (linear interpolation) between the values of the same pixels (if they fitted the required quality) of the closest preceding and following images.

We used the Normalized Difference Vegetation Index (NDVI) at a spatial resolution of 250 metres measured by the MODIS sensor. For each survey and each village, the average 16-day NDVI during the two-week period including the survey and the two-week period preceding the survey was extracted in the buffer zone. TiSEG was used to interpolate missing data using the same method that was used with LST data.

Daily rainfall data used was derived from the Tropical Rainfall Measuring Mission (TRMM) and other Satellite Precipitation products (3B42 Version 6). TRMM daily rainfall data were cumulated for the 15 days preceding each mosquito collection and spatially interpolated (using a kriging technique). The mean estimate of the cumulated rainfall in the buffer zone of each village was extracted. Cumulated rainfall was expressed in millimetres. The same procedure was used to extract the number of rainy days over the 15 days preceding each mosquito collection in each village.

### Spatial only data

A Digital Elevation Model (DEM) derived from the Satellite Pour l'Observation de la Terre (SPOT) SPOTDEM product with a 30 meters resolution was used to compute mean elevation, mean slope, count of sink and mean theoretical flow accumulation (i.e. for each pixel, the surface of its drainage area giving the theoretical drainage network capacity). To describe the ability of soils to accommodate for breeding sites, hydromorphic soils were identified on a digitized soil map of south Benin [46]. Hydromorphic soils are poorly-drained and characterized by an excess of water due to temporary waterlogging or the presence or rise of groundwater [47,48]. The percentage of area of hydromorphic soils in the buffer areas was computed.

A land-cover map was obtained by carrying out a supervised object-oriented nearest-neighbour classification [49] of a SPOT-5 satellite image (pixel of five metres) acquired on 01/23/2010 (eCognition™ software, Definiens-imaging, Munich, Germany). More than 140 plots of known land-cover were identified and geolocated in the field. These plots were used as training plots to define the feature of each land-cover class and then classify the overall image using a nearest-neighbour algorithm. The accuracy of the final classification was assessed using a confusion matrix to compare the allocated land cover class to 70 supplementary ground-truth validation plots.

A landscape analysis was conducted on the land-cover map to characterize its spatial structure. Using the Fragstats freeware [50], the following metrics were calculated for each class of land-cover in the buffer zone of each village: the percentage of the surface, the number of patches, the edge length. Other metrics were used to describe the diversity present within the whole landscape (i.e. for the entire buffer regardless of the class): the Patch Richness Density, the Simpson's Diversity Index and the Modified Simpson's Eveness Index (all metrics are defined in the Fragstats documentation [50]).

Market gardening could create suitable breeding sites due to the irrigation and water storage. However, these activities are difficult to discriminate on SPOT imagery because of the small size of fields. We therefore carried out field surveys (in March 2011) to inventory market gardening areas. Roads were digitalized from 1/50000 maps from 1968 available at the Institut Géographique National of Benin and updated using the SPOT-5 image.

In order to describe attractiveness and penetrability for malaria vectors, village perimeters were extracted by on screen digitalization using visual interpretation of the SPOT image. Then, area and population density were computed and the distance from each collection site to the perimeter of the village was measured. The number of neighbourhoods (homogenous groups of houses separated from other groups by a vegetated strip) was recorded to describe the clustered or spread-out layout of the villages. Using the Shape Metrics Tool (http://clear.uconn.edu/tools/Shape_Metrics/), the normalized Spin and Depth indices describing the shape of the villages were computed. Cattle farms were inventoried because they can interfere with host seeking behaviour of malaria vectors that may bite on cattle rather than humans [51].

### Statistical analysis

All analyses were performed using the 'R' software [52] and the additional 'lme4' [53] and 'pROC' [54] packages.

The probability of human-vector contact (PHVC) for each species and molecular forms considered during a survey (two consecutive nights) was assessed using a Binomial Mixed-Effect (BME) model with nested random effects at the village and collection site level. The dependent variable was the presence/absence of vectors collected during 1, 216 catches (19 villages * four collection sites * two places (indoors and outdoors) * eight surveys). The BME was adjusted for the vector control intervention and the collector's position (indoor or outdoor). The independent variables were tested both as continuous and categorical variables (after recoding). They were kept as continuous variables whenever possible, and were stratified into terciles (or into presence/absence if more relevant) when necessary to accommodate for non-monotonic and non-linear responses. Only variables found to be significantly correlated (p<0.05) with the PHVC of malaria vector species were kept for analysis of collinearity. Collinear covariates were deleted based on empirical knowledge until the Variance Inflation Factor (VIF) of remaining variables was below three [55].

All selected variables from the univariate analysis were introduced in the multivariate BME models, and a backward procedure was applied to select only those that remained significant with a p-value<0.05 in the final model.

The structure of the models was evaluated by 8-fold cross-validation with the data of each survey successively used for validation. The predictive accuracy of the models was assessed using the ROC (Receiver Operating Characteristic) curve method [56]. The area under the curve (AUC) of the ROC curve, its 95% confidence interval (95% CI), sensitivity and specificity were calculated to assess the quality of the models [54].

### Risk maps of human-vector contact

Based on the final multivariate BME models, two seasonal maps of the risk of contact with the three species were computed for the 15-16/01/2009 (dry season) and the 30-31/06/2009 (rainy season). Covariates describing attractiveness and penetrability for which data were not available at all points of the area were set at a constant value equal to the mean calculated for overall villages.

### Ethics statement

The IRD (Institut de Recherche pour le Développement) Ethics Committee and the National Research Ethics Committee of Benin approved the study (CNPERS, reference number IRB00006860). All necessary permits were obtained for the described field studies. No mosquito collection was carried out without the approval of the head of the village, the owner and occupants of the collection house. Mosquito collectors gave their written informed consent and were treated free of charge for malaria presumed illness throughout the study.

## Results

### Malaria vector collection

In the 19 villages (Table 1) during 2,432 human-nights of collection, 2,379 malaria vectors were collected: 1,091 were *An. funestus* and 1,288 were *An. gambiae s.s.* among which, 1,063 belonged to the molecular M form and 225 belonged to the molecular S form. Over 1,216 catches (two human-nights per site per survey) were used for modelling, only 252, 323, and 114 were positive for *An. funestus*, *An. gambiae* M form, and *An. gambiae* S form, respectively.

**Table 1:** Spatial coordinates of the studied villages and entomological data

	**Village center coordinates**		**Proportion (%) of catches positive for**			**Total no. of vectors collected**		
**Village name**	**Latitude**	**Longitude**	***An. funestus***	***An. gambiae *M form**	***An. gambiae *S form**	***An. funestus***	***An. gambiae *M form**	***An. gambiae *S form**
Agouako	N 06°30'30.2"	E 002°09'47.7"	1.56	9.38	6.25	1	7	5
Amoulehoue	N 06°26'36.3"	E 002°07'19.9"	35.94	26.56	4.69	50	53	8
Assogbenou daho	N 06°27'19.5"	E 002°05'24.5"	4.69	18.75	6.25	3	22	6
Ayidohoue	N 06°31'35.8"	E 002°05'59.6"	1.56	6.25	0.00	1	8	0
Adjahassa	N 06°33'11.3"	E 002°09'34.5"	0.00	18.75	6.25	0	27	4
Dokanmey	N 06°33'13.9"	E 002°13'37.5"	3.13	10.94	4.69	2	9	5
Kindjitokpa	N 06°25'37.5"	E 002°07'31.3"	84.38	39.06	6.25	297	127	6
Tokoli-vidjinnagnimon	N 06°26'57.1"	E 002°09'36.6"	65.63	34.38	12.50	230	81	10
Hekandji	N 06°29'26.0"	E 002°05'51.0"	0.00	4.69	6.25	0	3	5
Hla	N 06°32'34.0"	E 002°11'54.8"	3.13	21.88	10.94	2	41	7
Hounkponouhoue	N 06°33'31.4"	E 002°05'27.7"	1.56	7.81	15.63	2	8	12
Manguevier	N 06°28'21.8"	E 002°09'29.3"	18.75	53.13	4.69	18	95	4
Tanto	N 06°33'13.1"	E 002°10'48.0"	0.00	29.69	23.44	0	88	64
Lokohoue	N 06°24'24.2"	E 002°10'32.1"	71.88	53.13	7.81	273	175	10
Todo	N 06°30'09.9"	E 002°08'09.2"	0.00	26.56	6.25	0	56	6
Tokoli-dozouzrame	N 06°27'35.2"	E 002°10'17.1"	50.00	43.75	25.00	112	100	22
Wanho	N 06°27'32.9"	E 002°11'19.6"	46.88	62.50	0.00	96	77	0
Agadon	N 06°33'35.8"	E 002°06'25.7"	3.13	23.44	25.00	2	67	43
Zoume	N 06°32'42.7"	E 002°06'50.5"	1.56	14.06	6.25	2	19	8

Latitudes and longitudes are given in the standard coordinate system WGS84. Proportion of catches positive for each species was calculated over 64 catches carried out in each village (4 collection sites * 2 places (indoors and outdoors) * 8 repetitions (surveys)).

### Land-cover map

**Figure 1:**
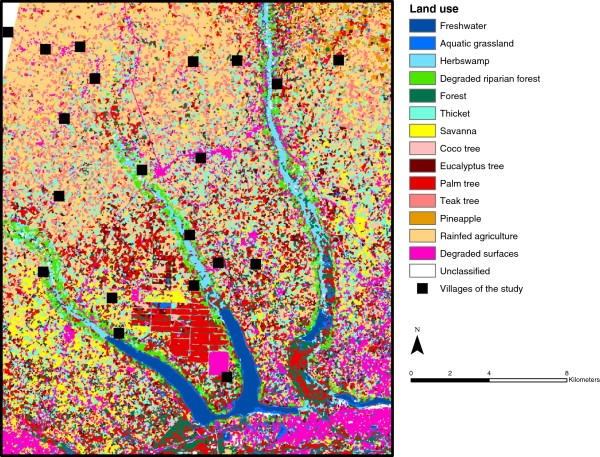
**Land-cover map based on a supervised object-oriented classification of a SPOT-5 satellite image acquired on 01/23/2010.** This map was obtained by carrying out a supervised object-oriented nearest-neighbour classification using the software eCognition™ (Definiens-imaging, Munich, Germany). More than 210 plots of known land-cover were identified and geolocated in the field to be used as training and validation (ground-truth) data.

Fourteen land-cover classes were discriminated: surface freshwater (1.8% of the total surface), aquatic grassland (1.4%), herb swamp (2.1%), coco tree (1%), eucalyptus tree (4.5%), palm tree (11.5%), teak tree (6.3%), pineapple (1.8%), rainfed agriculture (28%), forest (6%), degraded riparian forest (3%), thicket (13.9%), savanna (5.9%), and degraded surface (bare soil and constructed areas, 12.4%). The confusion matrix revealed that the overall accuracy of the classification was excellent (98.9%). The resulting land-cover map of the study area is presented in Figure 1.

### Determinants of the probability of human-vector contact

Covariates that were kept in final multivariate models, their odds-ratio and 95% confidence intervals are presented in Tables 2 (*An. funestus*), Table 3 (*An. gambiae* M form), and Table 4 (*An. gambiae* S form).

**Table 2:** **Multivariate binomial mixed-effect model of the risk of human-*An. funestus *contact**

		**OR**	**95% CI**		**P-value**	
Surface water	absence	1				
	presence	228.775	81.888	639.144	< 2e-16	***
Area of hydromorphic soils (per additional 100 ha)		3.823	2.136	6.843	< 5e-4	***
NDVI 2 weeks before the catch		0.001	0.000	0.067	0.001	***
Diurnal temperature 2 weeks before the catch (per additional °C)		1.321	1.175	1.485	< 5e-4	***
Nocturnal temperature (1 week before the catch; in °C)	<20.68	1				
	20.68-21.64	0.090	0.040	0.200	< 5e-4	***
	≥21.64	0.129	0.053	0.316	< 5e-4	***
Cumulated precipitation 16 days preceding the catch (per additional mm)		1.012	1.009	1.016	< 5e-4	***
Number of neighbourhoods	<2	1				
	2	2.187	0.767	6.233	0.143	
	≥3	116.273	35.779	377.856	< 5e-4	***
Vector control intervention	TLLIN	1				
	ULLIN	0.679	0.311	1.481	0.330	
	ULLIN+CTPS	0.264	0.108	0.646	0.004	**
	TLLIN+IRS	0.398	0.151	1.045	0.061	.

OR: Odds-ratio; CI: Confidence Interval; ha: hectares; °C: degrees Celsius; mm: millimetres; m: metres; TLLIN: Targeted distribution of Long-Lasting Insecticidal Nets; ULLIN: Universal distribution of LLIN; CTPS: Carbamate Treated Plastic Sheeting; IRS: carbamate Indoor Residual Spraying.

**Table 3:** **Multivariate binomial mixed-effect model of the risk of human-*An. gambiae *M form contact**

		**OR**	**95% CI**		**P-value**	
Proportion of unvegetated land (per additional %)		1.080	1.047	1.113	< 5e-4	***
Number of patches of unvegetated lands	<75	1				
	75-92	6.321	3.472	11.509	< 5e-4	***
	≥92	1.174	0.421	3.275	0.759	
Herbswamp	absence	1				
	presence	0.276	0.135	0.565	< 5e-4	***
Area of hydromorphic soils (per additional 100 ha)		20.323	9.908	41.688	< 2e-16	***
Domestic breeding sites	absence	1				
	presence	2.021	1.215	3.360	0.007	**
NDVI (during collection)	<0.591	1				
	0.591-0.652	2.472	1.427	4.281	0.001	**
	≥0.652	3.042	1.643	5.635	< 5e-4	***
Diurnal temperature 1 week before the catch (per additional °C)		0.735	0.614	0.882	0.001	***
Nocturnal temperature the week of the catch (per additional °C)		0.755	0.651	0.875	< 5e-4	***
Nocturnal temperature 2 weeks before the catch (per additional °C)		0.835	0.714	0.977	0.024	*
Number of days with rainfall during the 16 days preceding the catch (per additional day)		0.842	0.751	0.943	0.003	**
Cumulated precipitation during the 16 days preceding the catch (per additional mm)		1.020	1.015	1.025	< 5e-4	***
Number of cattle (per additional individual)		0.990	0.981	0.998	0.021	*
Number of neighbourhoods	<2	1				
	2	0.779	0.380	1.595	0.495	
	≥3	10.644	3.114	36.386	< 5e-4	***
Vector control intervention	TLLIN	1				
	ULLIN	3.214	1.241	8.324	0.016	*
	ULLIN+CTPS	1.902	0.820	4.416	0.134	
	TLLIN+IRS	0.459	0.170	1.239	0.124	

OR: Odds-ratio; CI: Confidence Interval; ha: hectares; °C: degrees Celsius; mm: millimetres; m: metres; TLLIN: Targeted distribution of Long-Lasting Insecticidal Nets; ULLIN: Universal distribution of LLIN; CTPS: Carbamate Treated Plastic Sheeting; IRS: carbamate Indoor Residual Spraying.

**Table 4:** **Multivariate binomial mixed-effect model of the risk of human-*An. gambiae *S form contact**

		**OR**	**95% CI**		**P-value**	
Road length (m)	<18,000	1				
	18,000 - 22,000	6.272	2.321	16.949	< 5e-4	***
	≥22,000	2.978	1.202	7.380	0.018	*
Number of patches of pineapple	<10	1				
	10-25	5.668	1.135	28.309	0.035	*
	≥25	1.581	0.664	3.763	0.301	
Elevation (per additional m)		1.058	1.028	1.088	< 5e-4	***
Slope (per additional degree)		0.551	0.291	1.042	0.067	.
Hydromorphic soil	absence	1				
	presence	13.407	4.705	38.206	< 5e-4	***
Nocturnal temperature 2 weeks before the catch (per additional °C)		1.330	1.086	1.628	0.006	**
NDVI 2 weeks before catch		0.016	0.000	0.639	0.028	*
Cumulated precipitation 16 days preceding the catch (per additional mm)		1.005	1.002	1.009	0.003	**
Number of breeding sites per 100 houses (per additional site)		1.055	0.999	1.114	0.056	.
Domestic breeding sites	absence	1				
	presence	2.668	1.485	4.792	0.001	**
Vector control intervention	TLLIN	1				
	ULLIN	2.799	1.016	7.709	0.046	*
	ULLIN+CTPS	1.016	0.365	2.824	0.976	
	TLLIN+IRS	0.615	0.152	2.483	0.495	
Collection site	Indoor	1				
	Outdoor	1.519	0.992	2.326	0.054	.

OR: Odds-ratio; CI: Confidence Interval; ha: hectares; C: degrees Celsius; mm: millimetres; m: metres; TLLIN: Targeted distribution of Long-Lasting Insecticidal Nets; ULLIN: Universal distribution of LLIN; CTPS: Carbamate Treated Plastic Sheeting; IRS: carbamate Indoor Residual Spraying.

Cumulated rainfall during the 15 days preceding the catch positively correlated to the PHVC of each vector species (p< 0.001), whereas the number of rainy days was negatively associated with the PHVC of *An. gambiae* M form. NDVI was correlated negatively with the PHVC of *An. gambiae* S form and *An. funestus* (NDVI two weeks before collection) but positively with *An. gambiae* M form (NDVI of the collection period).

The PHVC of the three species was explained by temperature data recorded two weeks before the catch. The PHVC of *An. funestus* and *An. gambiae* M form decreased respectively with the diurnal and the nocturnal temperatures recorded during the week or the week preceding the mosquito collection. They were also both positively correlated with the number of neighbourhoods.

PHVC of M and S forms of *An. gambiae* was positively correlated with the presence and the number of domestic breeding sites. Interestingly, the PHVC of the *An. gambiae* M form was associated with both the surface and an intermediate number of patches of unvegetated land but negatively correlated with the presence of herbswamp. The PHVC of *An. gambiae* M form decreased with the number of cattle recorded around the village.

The PHVC of *An. gambiae* S form was positively associated with the elevation, the length of roads, an intermediate number of patches of pineapple plantation (between 10 and 25) but negatively correlated with the slope. The presence of hydromorphic soils correlated with the PHVC of the three species and the surface of freshwater increased the PHVC of *An. funestus*.

The PHVC of *An. gambiae* S form was lower in the villages where ULLIN+CTPS were implemented compared to villages belonging to the reference group (TLLIN). However, the PHVC of *An. gambiae* M form or S form was higher in villages where ULLIN was implemented. The PHVC of *An. gambiae* S form was significantly higher outdoors than indoor.

### Validation

**Figure 2:**
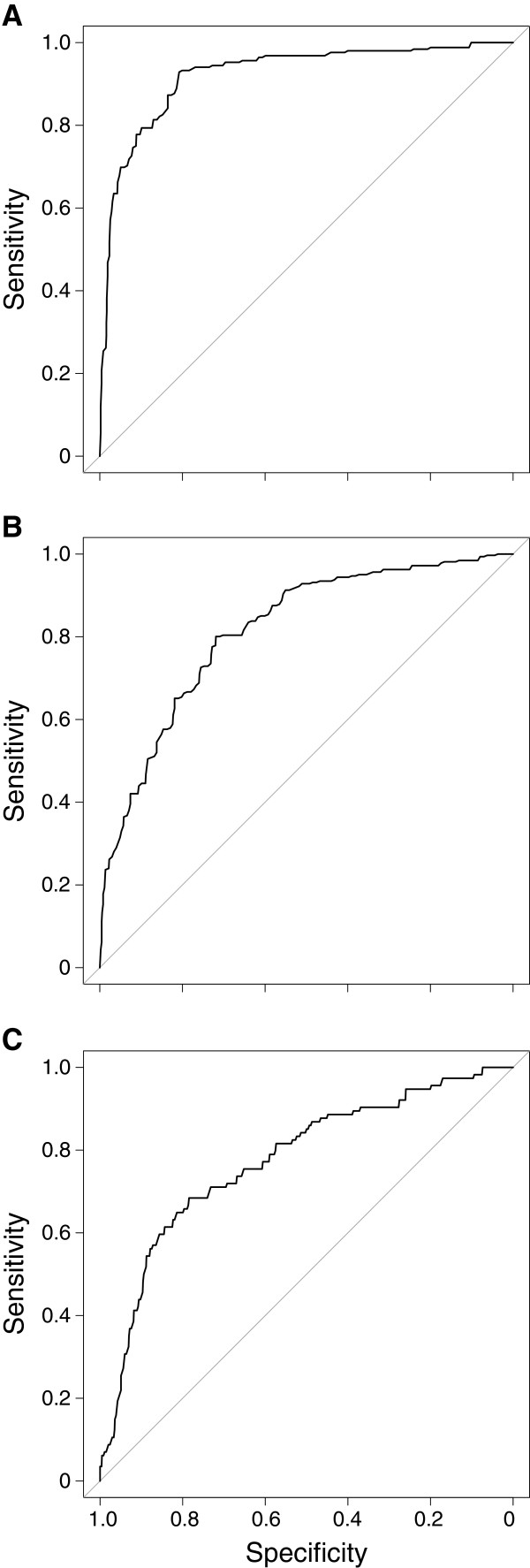
**ROC curve of the predictive model of the contact between human and (A) *An. funestus *(B) *An. gambiae *M form and (C) *An. gambiae *S form.** ROC (Receiver Operating Characteristic) curves were performed based on 8-fold cross-validations of the final multivariate models with the data of each survey successively used for validation. The curves plot the sensitivity versus one minus the specificity. Greater is the area under the curve, greater is the predictive accuracy of the model.

The ROC curves of the 8-fold cross validation are presented in Figure 2. The AUC of the *An. funestus* BME was 0.92 (95% CI 0.90-0.94; Figure 2A) indicating an excellent prediction. For the probability threshold 0.13, specificity was 80% (95% CI 78–83) and sensitivity was 93% (95% CI 90–96).

**Figure 3:**
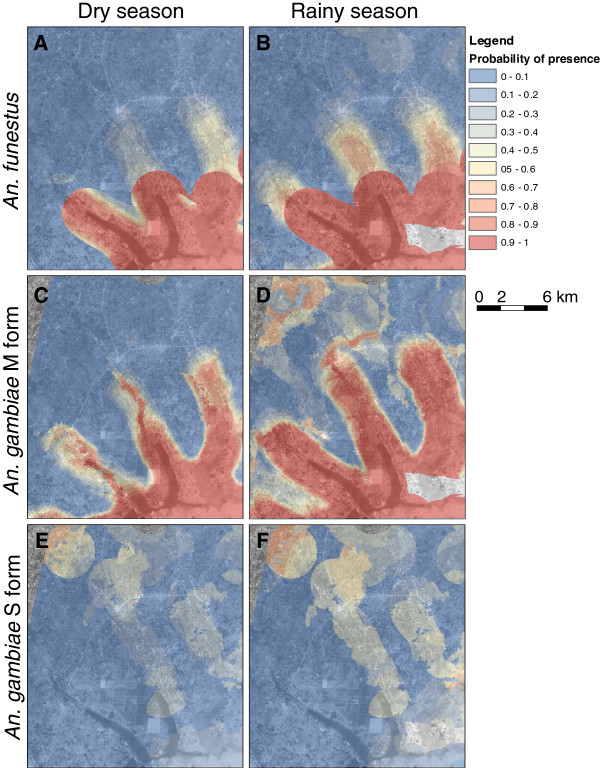
**Risk maps of the contact between human and (A, B) *An. funestus*, (C, D) *An. gambiae *M form and (E, F) *An. gambiae *S form for 2 nights during the dry and the rainy season.** Risk maps were computed based on the final multivariate binomial mixed-effect models. Two seasonal maps of the probability of contact with the three species/forms were produced for the 15-16/01/2009 (dry season) and the 30-31/06/2009 (rainy season). Covariates describing attractiveness and penetrability for which data were not available at all points of the area were set at a constant value equal to the mean calculated for overall villages.

The AUC of the *An. gambiae* M form BME was 0.84 (95% CI 0.81-0.86; Figure 2B) indicating a "good" prediction. For the probability threshold 0.21, specificity was 65% (95% CI 63–69) and sensitivity was 86% (95% CI 83–90). The AUC of the *An. gambiae* S form BME was 0.77 (95% CI 0.72-0.82; Figure 3C) indicating an "acceptable" prediction. For the probability threshold 0.12, specificity was 81% (95% CI 79–83) and sensitivity was 65% (95% CI 56–74).

### Risk maps

Maps of the predicted PHVC of *An. funestus*, *An. gambiae* M form, and *An. gambiae* S form for two nights during the dry and the rainy season are presented in Figure 3. During the dry season, *An. funestus* was highly concentrated around Toho Lake in the southern part of the study area. During the rainy season, we observed an increase of its PHVC along the downstream parts of the rivers that drain into Toho Lake and the lagoon. The PHVC of *An. gambiae* M form followed a similar distribution pattern in the dry season: areas of highest risk were found in the southern part of the study area, largely overlapping areas of high PHVC of *An. funestus*. Distribution of the PHVC of *An. gambiae* M form showed the most important seasonal variations spreading along the rivers and in the most elevated part of the study area during the rainy season. The PHVC of *An. gambiae* S form was lower than that of *An. funestus* and *An. gambiae* M form. Its distribution is very different as it is largely confined in the northern and eastern part of the study area. It represented a risk in the south only during the rainy season.

## Discussion

The M and S molecular forms of *An. gambiae s.s.* and *An. funestus* occurred in sympatry in the OKT region [31] and this setting is particularly favourable to study the environmental determinants involved in the spatial and temporal distribution of each malaria vector species. Here, we fitted specific models at an unprecedented level of resolution to predict the spatio-temporal risk of the human-vector contact in 19 villages in southern Benin. The models were validated using an 8-fold cross-validation technique. The predictions given by BME models were considered as "good" and "excellent" for *An. gambiae* M form and *An. funestus* respectively, whereas the predictions for the *An. gambiae* S form model was "acceptable". The lower predictive value of the *An. gambiae* S form model was likely due to the lower number of mosquitoes collected during the collection period. Indeed *An. gambiae* S form was previously described as being at the edge of its distribution area in southern Benin [57]. To our knowledge, this study is the one considering the most important number of environmental covariates likely to impact on the ecology of these malaria vectors. Indeed, we paid great attention to take into account most of the relevant factors driving the creation of breeding sites, the vector dispersion, and the vector survival. However, other factors (e.g. human settlements other than the studied villages, predation, personal protection, humidity, wind, etc.) affecting the contact between human and malaria vectors were not included in the analysis because of unavailability, poor quality, or low resolution.

The present study seems to indicate that *An. gambiae* M form was not dependent on the presence of permanent wetlands (freshwaters and aquatic grassland). This result contrasts with previous studies conducted in Burkina Faso and Cameroon [13,14]. Instead, we found a positive correlation between the PHVC of *An. gambiae* M form and both the surface and the number of patches of unvegetated land. This might be explained by the fact that the SPOT image used to assess land use was acquired during the dry season when seasonal wetlands were almost absent. The PHVC of *An. gambiae* M form was negatively correlated to the number of rainy days hence suggesting that this species may be particularly sensitive to flush-out. Nevertheless, all species were positively associated with the surface and presence of hydromorphic soils that might provide suitable semi-permanent breeding sites during the rainy season. As expected, the PHVC of *An. funestus* was predicted by the presence of freshwater bodies that could provide ideal breeding sites for this species [58].

Length of roads in the buffer area around the villages positively predicted the PHVC of *An. gambiae* S form. Indeed, road ditches and puddles created by vehicles are known to provide good breeding sites for this species, particularly during the rainy season [59]. Interestingly, only the intermediate class of number of patches (10 to 25) of pineapple plantations increased the probability of contact with *An. gambiae* S form. Leaf axils of pineapple plants could provide breeding sites for *An. gambiae* as previously observed in the neighbouring Nigeria [60] and regular traffic of vans carrying fruits could create ruts in roads. However, the relationship was not linear and not related to the surface indicating that external factors probably alter the development of *An. gambiae* S form when the number of plantations increases. We presume that different species of pineapples, cropping techniques or the presence of more or less predators may impact on this trend. Despite a small range of elevations and slopes in the study area, these factors were significantly associated with the PHVC of *An. gambiae* S form as described elsewhere in Africa [13-15]. *An. funestus* and *An. gambiae* M form were correlated to the number of neighbourhoods that is an indicator of the villages' layout. This may indicate that scattered habitations in a village might increase the attractiveness of the village for these species. This suggests that the field of attraction of a scattered village may be larger than that of a clustered village of the same size.

Temperatures recorded two weeks before the catch (corresponding to the larval period) explained the PHVC of the three species. The PHVC of *An. funestus* and *An. gambiae* S form was higher when temperatures increased. A different scenario was observed with *An. gambiae* M form: the lower the temperature (over different time periods comprised between the week of the catch and two weeks before the catch), the higher the PHVC of *An. gambiae* M form. This result could indicate that the M form was more susceptible to high water temperatures that can induce high mortality rates at the larval stage [61-63] and more active when temperatures were the lower. However, there is no published data to support these assumptions and the real underlying reason for these associations remains unclear. Our models show that the relationship between temperature and distribution of malaria vectors is complex. Indeed, both diurnal and nocturnal temperatures, over different time periods (comprised between the week of the catch and two weeks before the catch), were significantly predictive for two of the three species studied. Temperature is known to influence many aspects of the mosquito lifecycle such as larval growth, survival of adults and biting activity. Moreover, our team found a negative correlation between elevated temperature and LLIN use in Benin that could also impact on mosquito longevity and density [64]. Further investigations are needed to better understand the impact of temperature on the ecology of these vector species.

Our results indicate that the PHVC of the M and S molecular forms of *An. gambiae s.s.* was greater in presence of domestic breeding sites. This is worrying because it might indicate that villagers “bred” malaria vectors in their villages and inside their houses and might therefore support part of the malaria transmission. Since the majority of containers involved as breeding sites were used for water storage, these breeding sites were not rain-dependent and remained full especially during the dry season. No information is available yet regarding the *Anopheles* species present in the domestic breeding sites. Larviciding may then represent a complementary strategy for targeting malaria vectors within semi-urbanized population centres in rural areas [65].

Regarding vector control interventions, the probability of contact between humans and *An. funestus* was reduced only in villages sharing the ULLIN+CTPS combined intervention. This trend might reflect a decrease in the density of *An. funestus* following the implementation of vector control intervention hence underlying a certain efficacy of the treatments. In contrast, the PHVC of both molecular forms of *An. gambiae s.s.* was higher in villages where ULLIN were implemented alone. This finding contrasts with that of Corbel and colleagues [5] as they did not find a significant difference between ULLIN and TLLIN in terms of mosquito density and malaria transmission. We assume that mosquito collectors were overexposed to the vector bite. Indeed, high levels of LLIN use as observed in the ULLIN arm [5] might have contributed to divert a part of the vector population to alternatives hosts (like cattle, as suggested by the *An. gambiae* M form model) or to unprotected hosts such as our collectors [51,66].

## Conclusion

To conclude, the predictive maps showed seasonal and spatial variations of the risk of human-vector contact for the all three vectors. These variations were characterized by an increase in the distribution areas of the three species during the rainy season. Such maps could help Malaria Control Programs to implement more effective vector control strategies by taking into account the dynamics of malaria vector species. The use of larvicides in domestic breeding sites might be a cost effective strategy for malaria control [67,68] considering the possible ecology of *An. gambiae s.s.* in rural areas in Benin.

## Competing interests

The authors declare that they have no competing interests.

## Authors’ contributions

NM, VC, FC and HG designed the study. NM, ASB, and AD collected field data. NM treated the data. NM and HG performed the analysis. NM, VC and HG wrote the article. All authors read and approved the final version of the article.

## References

[B1] Murray CJL Rosenfeld LC Lim SS Andrews KG Foreman KJ Haring D Fullman N Naghavi M Lozano R Lopez AD Global malaria mortality between 1980 and 2010: a systematic analysis Lancet 2012 379 413 431 10.1016/S0140-6736(12)60034-8 22305225

[B2] World Health Organization World Malaria Report 2011 2011 Geneva: World Health Organization 259

[B3] Feachem R Sabot O A new global malaria eradication strategy Lancet 2008 371 1633 1635 10.1016/S0140-6736(08)60424-9 18374409

[B4] Trape JF Tall A Diagne N Ndiath O Ly AB Faye J Dieye-Ba F Roucher C Bouganali C Badiane A Malaria morbidity and pyrethroid resistance after the introduction of insecticide-treated bednets and artemisinin-based combination therapies: a longitudinal study Lancet Infect Dis 2011 11 925 932 10.1016/S1473-3099(11)70194-3 21856232

[B5] Corbel V Akogbeto M Damien GB Djenontin A Chandre F Rogier C Moiroux N Chabi J Banganna B Padonou GG Henry MC Combination of malaria vector control interventions in pyrethroid resistance area in Benin: a cluster randomised controlled trial Lancet Infect Dis 2012 12 617 626 10.1016/S1473-3099(12)70081-6 22682536

[B6] Asidi A N'Guessan R Akogbeto M Curtis C Rowland M Loss of household protection from use of insecticide-treated nets against pyrethroid-resistant mosquitoes, benin Emerg Infect Dis 2012 18 1101 1106 10.3201/eid1807.120218 22709930 PMC3376816

[B7] Ferguson HM Dornhaus A Beeche A Borgemeister C Gottlieb M Mulla MS Gimnig JE Fish D Killeen GF Ecology: a prerequisite for malaria elimination and eradication PLoS Med 2010 7 e1000303 10.1371/journal.pmed.1000303 20689800 PMC2914634

[B8] The malERA Consultative Group on Vector Control A Research Agenda for Malaria Eradication: Vector Control PLoS Med 2011 8 e1000401 21311587 10.1371/journal.pmed.1000401 PMC3026704

[B9] Sinka ME Bangs MJ Manguin S Rubio-Palis Y Chareonviriyaphap T Coetzee M Mbogo CM Hemingway J Patil AP Temperley WH A global map of dominant malaria vectors Parasit Vectors 2012 5 69 10.1186/1756-3305-5-69 22475528 PMC3349467

[B10] Manguin S Carnevale P Mouchet J Coosemans M Julvez J Richard-Lenoble D Sircoulon J Biodiversity of malaria in the world 2008 Montrouge, France: John Libbey Eurotext

[B11] Cohuet A Harris C Robert V Fontenille D Evolutionary forces on Anopheles: what makes a malaria vector Trends Parasitol 2010 26 130 136 10.1016/j.pt.2009.12.001 20056485

[B12] Della Torre A Tu Z Petrarca V On the distribution and genetic differentiation of Anopheles gambiae s.s. molecular forms Insect Biochem Mol Biol 2005 35 755 769 10.1016/j.ibmb.2005.02.006 15894192

[B13] Simard F Ayala D Kamdem GC Pombi M Etouna J Ose K Fotsing JM Fontenille D Besansky NJ Costantini C Ecological niche partitioning between Anopheles gambiae molecular forms in Cameroon: the ecological side of speciation BMC Ecol 2009 9 17 10.1186/1472-6785-9-17 19460146 PMC2698860

[B14] Costantini C Ayala D Guelbeogo WM Pombi M Some CY Bassole IH Ose K Fotsing JM Sagnon N Fontenille D Living at the edge: biogeographic patterns of habitat segregation conform to speciation by niche expansion in Anopheles gambiae BMC Ecol 2009 9 16 10.1186/1472-6785-9-16 19460144 PMC2702294

[B15] Kelly-Hope L Lawson B Wilson M Boakye D De Souza D Environmental factors associated with the distribution of Anopheles gambiae s.s in Ghana; an important vector of lymphatic filariasis and malaria PLoS One 2010 5 e9927 10.1371/journal.pone.0009927 20360950 PMC2847902

[B16] Ndiath MO Cohuet A Gaye A Konate L Mazenot C Faye O Boudin C Sokhna C Trape JF Comparative susceptibility to Plasmodium falciparum of the molecular forms M and S of Anopheles gambiae and Anopheles arabiensis Malar J 2011 10 269 10.1186/1475-2875-10-269 21929746 PMC3184635

[B17] Gimonneau G Pombi M Choisy M Morand S Dabire RK Simard F Larval habitat segregation between the molecular forms of the mosquito Anopheles gambiae in a rice field area of Burkina Faso, West Africa Med Vet Entomol 2011 26 9 17 21501199 10.1111/j.1365-2915.2011.00957.x PMC3140611

[B18] Diabate A Dabire RK Heidenberger K Crawford J Lamp WO Culler LE Lehmann T Evidence for divergent selection between the molecular forms of Anopheles gambiae: role of predation BMC Evol Biol 2008 8 5 10.1186/1471-2148-8-5 18190719 PMC2217532

[B19] Diabate A Dabire RK Kim EH Dalton R Millogo N Baldet T Simard F Gimnig JE Hawley WA Lehmann T Larval development of the molecular forms of Anopheles gambiae (Diptera: Culicidae) in different habitats: a transplantation experiment J Med Entomol 2005 42 548 553 10.1603/0022-2585(2005)042[0548:LDOTMF]2.0.CO;2 16119542

[B20] Gimonneau G Bouyer J Morand S Besansky NJ Diabate A Simard F A behavioral mechanism underlying ecological divergence in the malaria mosquito Anopheles gambiae Behav Ecol 2010 21 1087 1092 10.1093/beheco/arq114 22476108 PMC2920295

[B21] Gimonneau G Pombi M Dabire RK Diabate A Morand S Simard F Behavioural responses of Anopheles gambiae sensu stricto M and S molecular form larvae to an aquatic predator in Burkina Faso Parasit Vectors 2012 5 65 10.1186/1756-3305-5-65 22463735 PMC3352179

[B22] Ranson H N'Guessan R Lines J Moiroux N Nkuni Z Corbel V Pyrethroid resistance in African anopheline mosquitoes: what are the implications for malaria control? Trends Parasitol 2011 27 91 98 10.1016/j.pt.2010.08.004 20843745

[B23] Moiroux N Gomez MB Pennetier C Elanga E Djenontin A Chandre F Djegbe I Guis H Corbel V Changes in Anopheles funestus Biting Behavior Following Universal Coverage of Long-Lasting Insecticidal Nets in Benin J Infect Dis 2012 206 1622 1629 10.1093/infdis/jis565 22966127

[B24] Russell TL Govella NJ Azizi S Drakeley CJ Kachur SP Killeen GF Increased proportions of outdoor feeding among residual malaria vector populations following increased use of insecticide-treated nets in rural Tanzania Malar J 2011 10 80 10.1186/1475-2875-10-80 21477321 PMC3084176

[B25] Govella NJ Ferguson H Why Use of Interventions Targeting Outdoor Biting Mosquitoes will be Necessary to Achieve Malaria Elimination Front Physiol 2012 3 199 22701435 10.3389/fphys.2012.00199 PMC3372949

[B26] Diabate A Baldet T Chandre C Dabire KR Kengne P Guiguemde TR Simard F Guillet P Hemingway J Hougard JM KDR mutation, a genetic marker to assess events of introgression between the molecular M and S forms of Anopheles gambiae (Diptera: Culicidae) in the tropical savannah area of West Africa J Med Entomol 2003 40 195 198 10.1603/0022-2585-40.2.195 12693848

[B27] Chandre F Manguin S Brengues C Dossou Yovo J Darriet F Diabate A Carnevale P Guillet P Current distribution of a pyrethroid resistance gene (kdr) in Anopheles gambiae complex from west Africa and further evidence for reproductive isolation of the Mopti form Parassitologia 1999 41 319 322 10697876

[B28] Fanello C Petrarca V Della Torre A Santolamazza F Dolo G Coulibaly M Alloueche A Curtis CF Toure YT Coluzzi M The pyrethroid knock-down resistance gene in the Anopheles gambiae complex in Mali and further indication of incipient speciation within An. gambiae s.s Insect Mol Biol 2003 12 241 245 10.1046/j.1365-2583.2003.00407.x 12752657

[B29] Santolamazza F Calzetta M Etang J Barrese E Dia I Caccone A Donnelly MJ Petrarca V Simard F Pinto J Della Torre A Distribution of knock-down resistance mutations in Anopheles gambiae molecular forms in west and west-central Africa Malar J 2008 7 74 10.1186/1475-2875-7-74 18445265 PMC2405802

[B30] Dabire KR Diabate A Djogbenou L Ouari A N'Guessan R Ouedraogo JB Hougard JM Chandre F Baldet T Dynamics of multiple insecticide resistance in the malaria vector Anopheles gambiae in a rice growing area in South-Western Burkina Faso Malar J 2008 7 188 10.1186/1475-2875-7-188 18817564 PMC2564969

[B31] Djenontin A Bio-Bangana S Moiroux N Henry MC Bousari O Chabi J Osse R Koudenoukpo S Corbel V Akogbeto M Chandre F Culicidae diversity, malaria transmission and insecticide resistance alleles in malaria vectors in Ouidah-Kpomasse-Tori district from Benin (West Africa): A pre-intervention study Parasit Vectors 2010 3 83 10.1186/1756-3305-3-83 20819214 PMC2941749

[B32] Brooke BD Kloke G Hunt RH Koekemoer LL Temu EA Taylor ME Small G Hemingway J Coetzee M Bioassay and biochemical analyses of insecticide resistance in southern African Anopheles funestus (Diptera: Culicidae) Bull Entomol Res 2001 91 265 272 10.1079/BER2001108 11587622

[B33] Dabire RK Namountougou M Sawadogo SP Yaro LB Toe HK Ouari A Gouagna LC Simard F Chandre F Baldet T Population dynamics of Anopheles gambiae s.l. in Bobo-Dioulasso city: bionomics, infection rate and susceptibility to insecticides Parasit Vectors 2012 5 127 10.1186/1756-3305-5-127 22721002 PMC3424103

[B34] Corbel V N'Guessan R Brengues C Chandre F Djogbenou L Martin T Akogbeto M Hougard JM Rowland M Multiple insecticide resistance mechanisms in Anopheles gambiae and Culex quinquefasciatus from Benin, West Africa Acta Trop 2007 101 207 216 10.1016/j.actatropica.2007.01.005 17359927

[B35] Djogbenou L Pasteur N Akogbeto M Weill M Chandre F Insecticide resistance in the Anopheles gambiae complex in Benin: a nationwide survey Med Vet Entomol 2011 25 256 267 10.1111/j.1365-2915.2010.00925.x 21155858

[B36] Djegbe I Boussari O Sidick A Martin T Ranson H Chandre F Akogbeto M Corbel V Dynamics of insecticide resistance in malaria vectors in Benin: first evidence of the presence of L1014S kdr mutation in Anopheles gambiae from West Africa Malar J 2011 10 261 10.1186/1475-2875-10-261 21910856 PMC3179749

[B37] Gillies M Coetzee M A supplement to the Anophelinae of Africa South of the Sahara (Afrotropical Region) 1987 Johannesburg, South Africa: South African Institute for Medical Research

[B38] Gillies M De Meillon B The Anophelinae of Africa South of the Sahara (Ethiopian Zoogeographical Region) Publication of the South Afr Inst Med Res 1968 54 1 343

[B39] Koekemoer LL Kamau L Hunt RH Coetzee M A cocktail polymerase chain reaction assay to identify members of the Anopheles funestus (Diptera: Culicidae) group AmJTrop Med Hyg 2002 66 804 811 10.4269/ajtmh.2002.66.804 12224596

[B40] Scott JA Brogdon WG Collins FH Identification of single specimens of the Anopheles gambiae complex by the polymerase chain reaction AmJTrop Med Hyg 1993 49 520 529 10.4269/ajtmh.1993.49.520 8214283

[B41] Favia G Lanfrancotti A Spanos L Siden-Kiamos I Louis C Molecular characterization of ribosomal DNA polymorphisms discriminating among chromosomal forms of Anopheles gambiae s.s Insect Mol Biol 2001 10 19 23 10.1046/j.1365-2583.2001.00236.x 11240633

[B42] Silver JB Sampling the Larval Population 2008 Ecology: In Mosquito 137 338

[B43] World Health Organization Yellow-fever panel: report on the first session 1950 Geneva: World Health Organization 12 14782506

[B44] Service MW Mosquito (Diptera: Culicidae) dispersal–the long and short of it J Med Entomol 1997 34 579 588 9439109 10.1093/jmedent/34.6.579

[B45] Colditz RR Conrad C Wehrmann T Schmidt M Dech S TiSeG: A flexible software tool for time-series generation of MODIS data utilizing the quality assessment science data set IEEE Trans Geosci Remote Sens 2008 46 3296 3308

[B46] Volkoff B Willaime P Carte pédologique de reconnaissance de la République Populaire du Bénin à 1/200 000: feuille de Porto-Novo 1976 Paris: ORSTOM 41

[B47] Aubert G La classification pédologique utilisée en France Classification des Sols 1965 Gand: Symposium International 25 56

[B48] Young A Tropical soils and soil survey 1976 Cambridge: Cambridge Univ. Press

[B49] Benz UC Hofmann P Willhauck G Lingenfelder I Heynen M Multi-resolution, object-oriented fuzzy analysis of remote sensing data for GIS-ready information ISPRS J Photogramm Remote Sens 2004 58 239 258 10.1016/j.isprsjprs.2003.10.002

[B50] McGarigal K Cushman S Ene E FRAGSTATS v4: Spatial Pattern Analysis Program for Categorical and Continuous Maps Book FRAGSTATS v4: Spatial Pattern Analysis Program for Categorical and Continuous Maps 2012 City: University of Massachusetts

[B51] Killeen GF Smith TA Exploring the contributions of bed nets, cattle, insecticides and excitorepellency to malaria control: a deterministic model of mosquito host-seeking behaviour and mortality Trans R Soc Trop Med Hyg 2007 101 867 880 10.1016/j.trstmh.2007.04.022 17631372 PMC1949412

[B52] R Development Core Team R: A Language and Environment for Statistical Computing 2010 Austria: R Foundation for Statistical Computing

[B53] Bates D Maechler Mlme4: Linear mixed-effects models using S4 classes 2009 R package version 0.999375-32

[B54] Robin X Turck N Hainard A Tiberti N Lisacek F Sanchez JC Muller M pROC: an open-source package for R and S+ to analyze and compare ROC curves BMC Bioinforma 2011 12 77 10.1186/1471-2105-12-77 PMC3068975 21414208

[B55] Zuur AF Ieno EN Elphick CS A protocol for data exploration to avoid common statistical problems Methods in Ecology and Evolution 2010 1 3 14 10.1111/j.2041-210X.2009.00001.x

[B56] Park SH Goo JM Jo CH Receiver operating characteristic (ROC) curve: practical review for radiologists Korean J Radiol 2004 5 11 18 10.3348/kjr.2004.5.1.11 15064554 PMC2698108

[B57] Djogbenou L Pasteur N Bio-Bangana S Baldet T Irish SR Akogbeto M Weill M Chandre F Malaria vectors in the Republic of Benin: Distribution of species and molecular forms of the Anopheles gambiae complex Acta Trop 2010 114 116 122 10.1016/j.actatropica.2010.02.001 20138819

[B58] Hamon J Biologie d'*Anopheles funestus* Biologie des anophèles d'AOF et d'AEF 1955 Paris: ORSTOM 6

[B59] Edillo FE Toure YT Lanzaro GC Dolo G Taylor CE Spatial and habitat distribution of Anopheles gambiae and Anopheles arabiensis (Diptera: Culicidae) in Banambani village, Mali J Med Entomol 2002 39 70 77 10.1603/0022-2585-39.1.70 11931274

[B60] Anosike JC Nwoke BE Okere AN Oku EE Asor JE Emmy-Egbe IO Adimike DA Epidemiology of tree-hole breeding mosquitoes in the tropical rainforest of Imo State, south-east Nigeria Ann Agric Environ Med 2007 14 31 38 17655174

[B61] Bayoh MN Lindsay SW Effect of temperature on the development of the aquatic stages of Anopheles gambiae sensu stricto (Diptera: Culicidae) Bull Entomol Res 2003 93 375 381 14641976 10.1079/ber2003259

[B62] Bayoh MN Lindsay SW Temperature-related duration of aquatic stages of the Afrotropical malaria vector mosquito Anopheles gambiae in the laboratory Med Vet Entomol 2004 18 174 179 10.1111/j.0269-283X.2004.00495.x 15189243

[B63] Lyimo EO Takken W Koella JC Effect of rearing temperature and larval density on larval survival, age at pupation and adult size of Anopheles gambiae Entomol Exp Appl 1992 63 265 271 10.1111/j.1570-7458.1992.tb01583.x

[B64] Moiroux N Boussari O Djènontin A Damien G Cottrell G Henry M-C Guis H Corbel V Dry Season Determinants of Malaria Disease and Net Use in Benin. West Africa PLoS One 2012 7 e30558 10.1371/journal.pone.0030558 22291987 PMC3264611

[B65] White MT Griffin JT Churcher TS Ferguson NM Basanez MG Ghani AC Modelling the impact of vector control interventions on Anopheles gambiae population dynamics Parasit Vectors 2011 4 153 10.1186/1756-3305-4-153 21798055 PMC3158753

[B66] Lines JD Myamba J Curtis CF Experimental hut trials of permethrin-impregnated mosquito nets and eave curtains against malaria vectors in Tanzania Med Vet Entomol 1987 1 37 51 10.1111/j.1365-2915.1987.tb00321.x 2979519

[B67] Fillinger U Lindsay SW Larval source management for malaria control in Africa: myths and reality Malar J 2011 10 353 10.1186/1475-2875-10-353 22166144 PMC3273449

[B68] Worrall E Fillinger U Large-scale use of mosquito larval source management for malaria control in Africa: a cost analysis Malar J 2011 10 338 10.1186/1475-2875-10-338 22067606 PMC3233614

